# Zinc as a Signal to Stimulate Red Blood Cell Formation in Fish

**DOI:** 10.3390/ijms18010138

**Published:** 2017-01-11

**Authors:** Yen-Hua Chen, Jhe-Ruei Shiu, Chia-Ling Ho, Sen-Shyong Jeng

**Affiliations:** 1 Center of Translational Medicine, Department of Basic Medicine, Xiamen Medical College, Xiamen 361023, China; yanhua09123@hotmail.com; 2 Department of Food Science, College of Life Sciences, National Taiwan Ocean University, Keelung 20224, Taiwan; syujhe.7@gmail.com (J.-R.S.); tina41048@gmail.com (C.-L.H.)

**Keywords:** zinc, erythropoiesis, red blood cells, fish, erythropoietin

## Abstract

The common carp can tolerate extremely low oxygen levels. These fish store zinc in a specific zinc-binding protein presented in digestive tract tissues, and under low oxygen, the stored zinc is released and used as a signal to stimulate erythropoiesis (red blood cell formation). To determine whether the environmental supply of zinc to other fish species can serve as a signal to induce erythropoiesis as in the common carp, head kidney cells of four different fish species were cultured with supplemental ZnCl_2_. Zinc stimulated approximately a three-fold increase in immature red blood cells (RBCs) in one day. The stimulation of erythropoiesis by zinc was dose-dependent. ZnSO_4_ solution was injected into an experimental blood loss tilapia model. Blood analysis and microscopic observation of the blood cells indicated that, in vivo, the presence of additional zinc induced erythropoiesis in the bled tilapia. In the fish species studied, zinc could be used as a signal to stimulate erythropoiesis both in vitro and in vivo. The present report suggests a possible approach for the induction of red blood cell formation in animals through the supply of a certain level of zinc through either diet or injection.

## 1. Introduction

In humans and other mammals, erythropoiesis, the process of red blood cell production, occurs in the bone marrow; in fish, the head kidney is the main erythropoietic organ [1]. The cellular composition of the teleost head kidney resembles that of mammalian bone marrow [2]. Erythropoiesis in fish is similar to that in other vertebrates [3,4]. Erythropoiesis in mammals is regulated by the hormone erythropoietin (EPO) [5,6]. Fish and mammalian erythropoietic systems have similar responses to hypoxia because erythropoiesis is influenced by EPO in fish [7]. The human hepatoma cell lines HepG2 and Hep3B have been utilized to study the regulation of EPO. Studies of Hep3B cells revealed that Co^2+^, Ni^2+^, and Mn^2+^ can stimulate EPO synthesis, whereas Zn^2+^ is ineffective [8]. However, Chen et al. [1] observed zinc stimulation of erythropoiesis in vitro when common carp head kidney cells were cultured with ZnCl_2_ supplementation. In vivo, an increase in the rate of erythropoiesis in the common carp was triggered and facilitated by zinc in the head kidney when the common carp were exposed to air [9]. In nature, stimulation of erythropoiesis in common carp by zinc occurs via the high concentration of zinc stored in a specific 43 kDa zinc-binding protein present in the digestive tract tissue of the common carp [10]. When needed, such as under conditions of anoxia, the zinc in the 43-kDa zinc-binding protein is released [11] and used as a signal to stimulate the formation of new red blood cells (RBCs) in the head kidney of the common carp [1,9]. It is of great interest to determine whether the supply of additional zinc to other fish species from the environment can serve as a signal to induce erythropoiesis as in the common carp. In this report, the effects of supplementation of ZnCl_2_ to cultures of the head kidney cells of four fish species were determined: (1) crucian carp, *Carassius carassius*; (2) grass carp, *Ctenopharyngodon idella*; (3) silver carp, *Aristichthys nobilis*; and (4) tilapia, *Oreochromis aureus*. In addition to this in vitro study, ZnSO_4_ solution was injected into an experimental blood loss tilapia model to determine whether zinc serves as a signal to stimulate erythropoiesis in fish in vivo. Our results indicated that zinc could be used as a signal to induce RBC formation in fish both in vitro and in vivo.

## 2. Results

### 2.1. Suspension Culture of the Head Kidney Cells from Four Fish Species with or without ZnCl_2_

**Figure 1:**
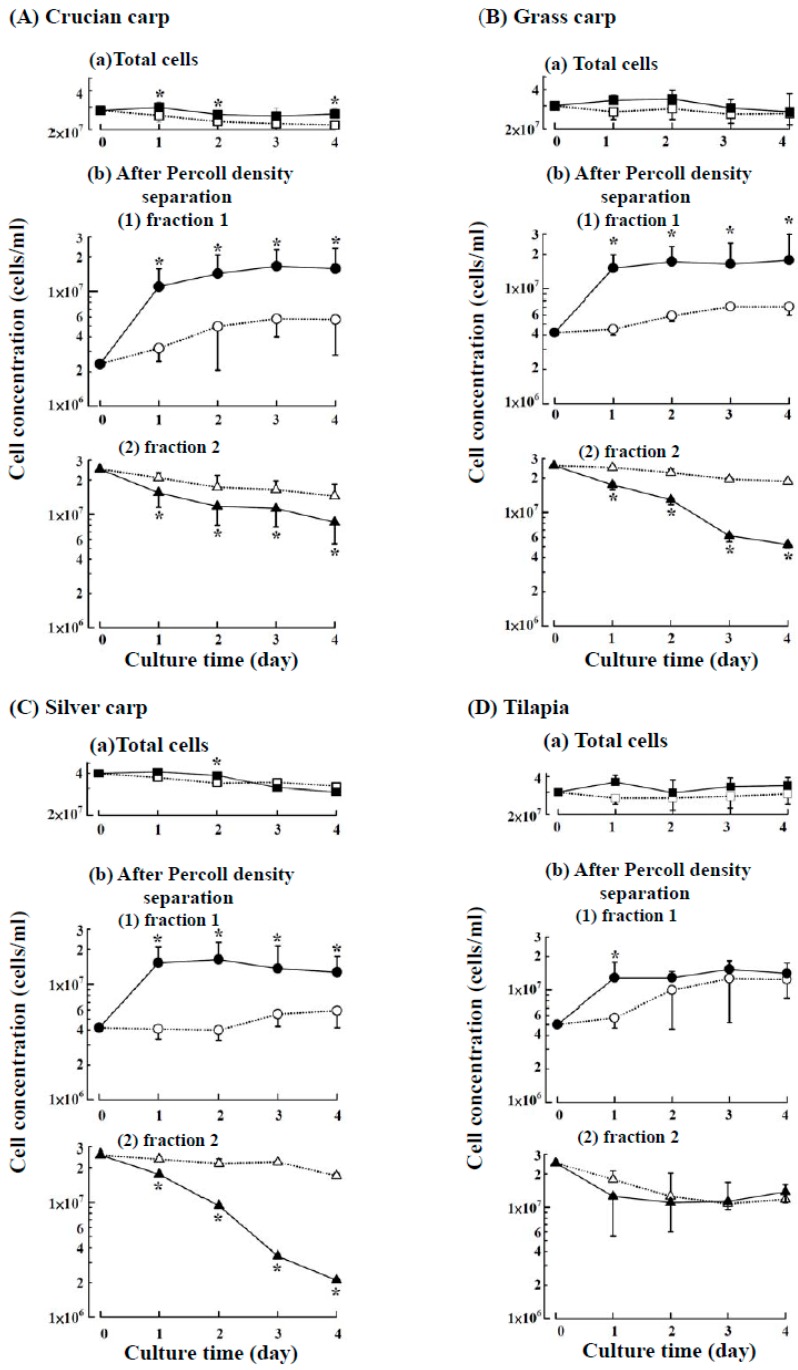
Suspension cultures of head kidney cells from the four fish species with or without ZnCl_2_ supplementation. (**A**) Crucian carp; (**B**) grass carp; (**C**) silver carp; and (**D**) tilapia. All cells remained in suspension for four days. The growth curve of the total cells is shown in (**a**). The total cells were further separated by Percoll density centrifugation into fractions 1 and 2 (*p* = 1.020 and 1.070 g/mL), and the growth curves are shown in **b**(**1**) and **b**(**2**), respectively. Filled symbols and continuous lines represent cultures supplemented with ZnCl_2_; open symbols and broken lines represent cultures without ZnCl_2_. The data are expressed as the means ± SDs of four independent experiments. * Statistically significant differences with *p* < 0.05 between the groups supplemented with or without ZnCl_2_.

In teleost fish, the maturation of erythrocytes involves a progressive increase in cell size [12]. For common carp, immature RBCs can be separated from mature RBCs through two-fraction separation with discontinuous Percoll density centrifugation. The immature RBCs are separated into fraction l (*p* = 1.020 g/mL) due to their smaller cell size, whereas the mature RBCs and other larger cells are separated into fraction 2 (*p* = 1.070 g/mL) [1]. The morphology of the head kidney cells of crucian carp, grass carp, silver carp and tilapia were found to be similar as in other species of fish, the major cells were lymphocytes, granulocytes, and erythrocytes [1,2,3,4]. After Percoll density centrifugation, it was found that the immature RBCs were separated into fraction 1 the same as that reported before [1]. As shown in Figure 1, after suspension culture of head kidney cells with or without ZnCl_2_, the total cell density changed only slightly during the four-day period for all four fish species (Figure 1A-a,B-a,C-a,D-a). However, an increase in the number of cells in fraction 1 and a decrease in the number of cells in fraction 2 were observed for all four fish species (Figure 1A-b,B-b,C-b,D-b). In fraction 1, for all four fish species, the increased cell density was significantly higher in the ZnCl_2_ groups than in the groups without ZnCl_2_ after day one and entered the plateau phase from day two to four (Figure 1A-b-1,B-b-1,C-b-1,D-b-1). At day one, the ratio of cell numbers in fraction l were approximately three-fold higher in the ZnCl_2_ groups than in groups without ZnCl_2_ for all four fish species. These results demonstrate that zinc stimulated the growth of the fraction 1 cells in all four fish species in one day, with an approximately three-fold increase in the proliferation of new immature RBCs.

### 2.2. Effects of ZnCl_2_ Levels on the Growth of Fraction 1 Cells

**Figure 2:**
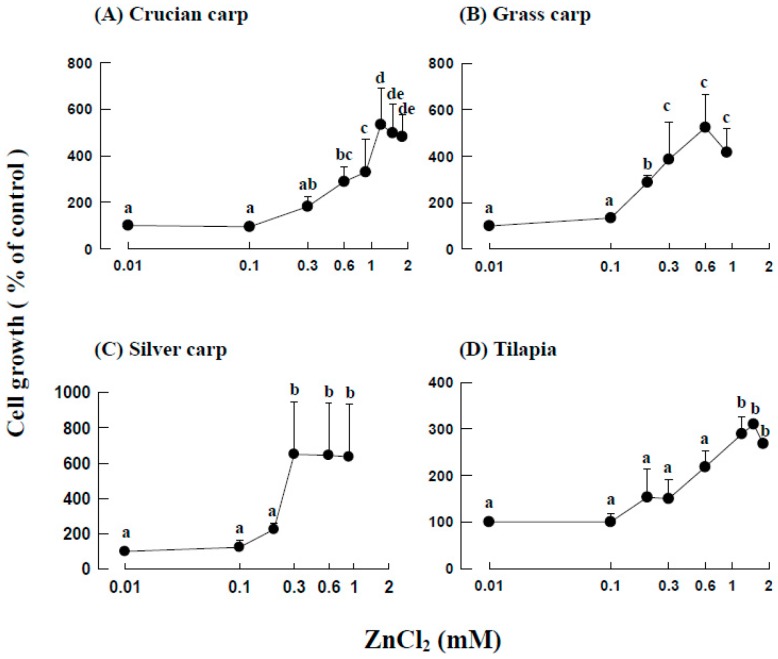
Effects of ZnCl_2_ concentrations on erythropoiesis in fish head kidney cells: (**A**) crucian carp, (**B**) grass carp, (**C**) silver carp, and (**D**) tilapia. The fish head kidney cells were suspension cultured with 0.01 (control), 1.0, or 1.8 mM ZnCl_2_ in the presence of 10% fish serum. Measurement of the cell growth is described in Materials and Methods. The results are the means ± SDs of six independent experiments. Values with different letter superscripts are significantly different at *p* < 0.05.

For crucian carp, grass carp, silver carp, and tilapia, significant activation of fraction 1 cell growth was observed at 0.6, 0.2, 0.3, and 1.2 mM ZnCl_2_, and maximal cell growth was approximately 500%, 520%, 660%, and 290%, respectively (Figure 2).

### 2.3. Characteristics of the Cultured Head Kidney Cells of the Crucian Carp

**Figure 3:**
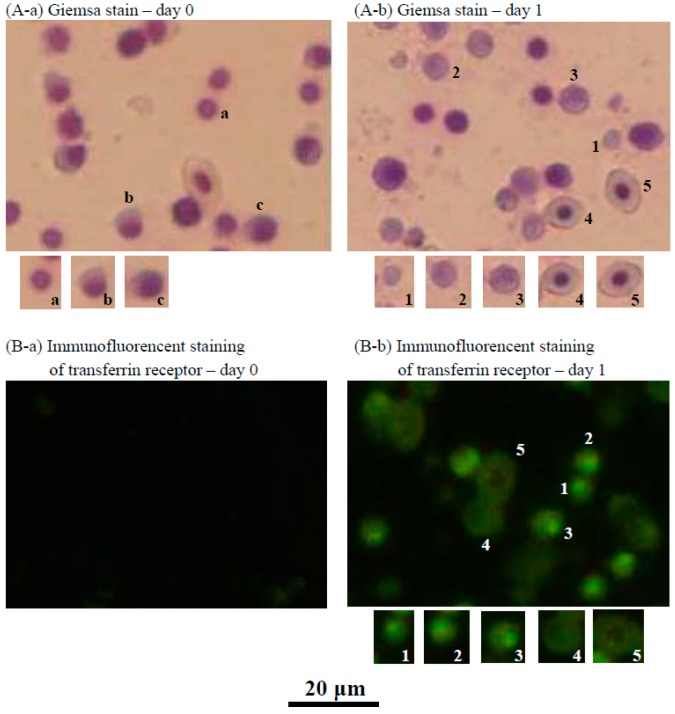
Cells collected from the suspension cultures of crucian carp head kidney cells grown in medium supplemented with ZnCl_2_. (**A**-**a**) Giemsa-stained cells collected at day zero. The major cells were lymphocytes (**a**) (approximately 70%); other cells, including neutrophilic progranulocytes (**b**) and basophilic granulocytes (**c**) were also observed; (**A**-**b**) Giemsa-stained cells collected at day one. Various newly proliferated cells (approximately 42%) were identified as erythrocytes of different development stages: 1. lymphoid hemoblasts, 2. early erythroblasts, 3. polychromatophilic erythroblasts, 4. orthochromatic erythroblasts, and 5. erythrocytes; (**B**-**a**) Immunofluorescent staining of the transferrin receptor of the cells at day zero; no cells were stained; and (**B**-**b**) Immunofluorescent staining of the transferrin receptor of the cells at day one. Comparing the cell size and cell morphology in (**A**-**b**) and (**B**-**b**), the transferrin receptor immunofluorescent-stained cells were RBC stages 1, 2, and 3. RBC stages 4 and 5 and cells other than RBCs were not immunofluorescently stained.

As shown in Figure 3A-b, when crucian carp head kidney cells were cultured with supplemental ZnCl_2_ for one day, many new cells proliferated that were identified as erythrocytes at different stages of development. Immunofluorescence staining of the transferrin receptor was only observed in RBCs at stages 1, 2, and 3, and not in RBCs at stages 4 and 5 (Figure 3B-b). This result indicates that immunofluorescence staining of the transferrin receptor can be used to identify immature RBCs (RBC stages 1, 2, and 3).

### 2.4. Characteristics of the Cultured Head Kidney Cells of Grass Carp, Silver Carp and Tilapia

**Figure 4:**
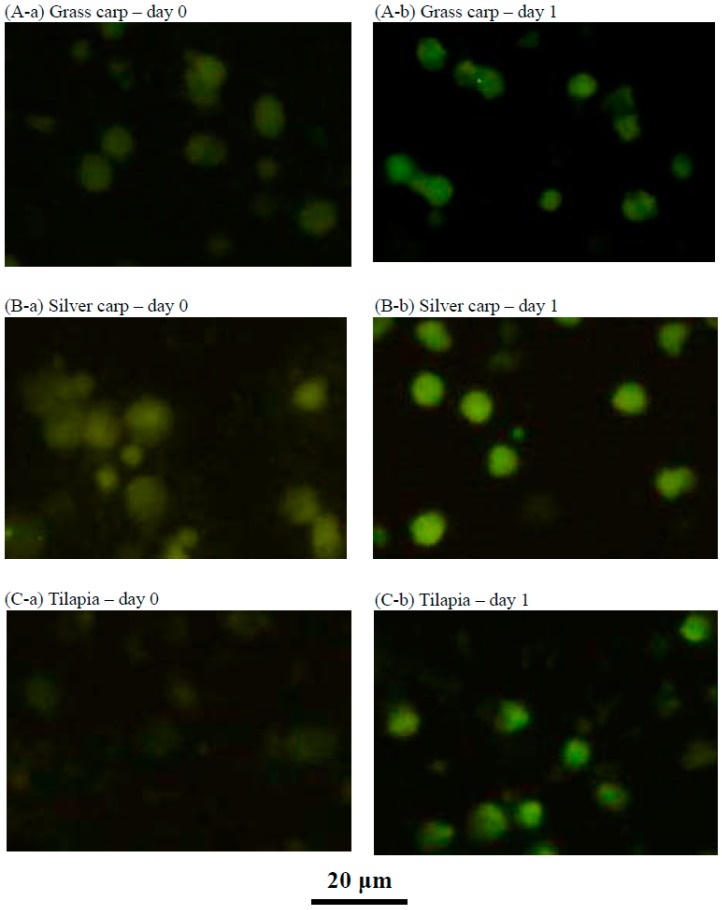
Immunofluorescent staining of the transferrin receptor of cells collected from suspension cultures of head kidney cells from (**A**) grass carp, (**B**) silver carp, and (**C**) tilapia at day zero and day one. At day 0, virtually no cells were immunofluorescently stained in the three fish species, as shown in (**A**-**a**), (**B**-**a**), and (**C**-**a**). At day one, various cells were immunofluorescently stained, representing newly proliferated RBC cells in stages 1, 2, and 3, as shown in (**A**-**b**), (**B**-**b**), and (**C**-**b**).

Immunofluorescent staining of the transferrin receptor revealed the proliferation of new immature RBCs when the three fish species (grass carp, silver carp, and tilapia) were supplemented in culture with ZnCl_2_ for one day (Figure 4).

### 2.5. Isolation of the Active Substance in Fish Serum That Stimulates the Proliferation of Fraction 1 Cells in the Head Kidney Cells of the Four Fish Species

**Figure 5:**
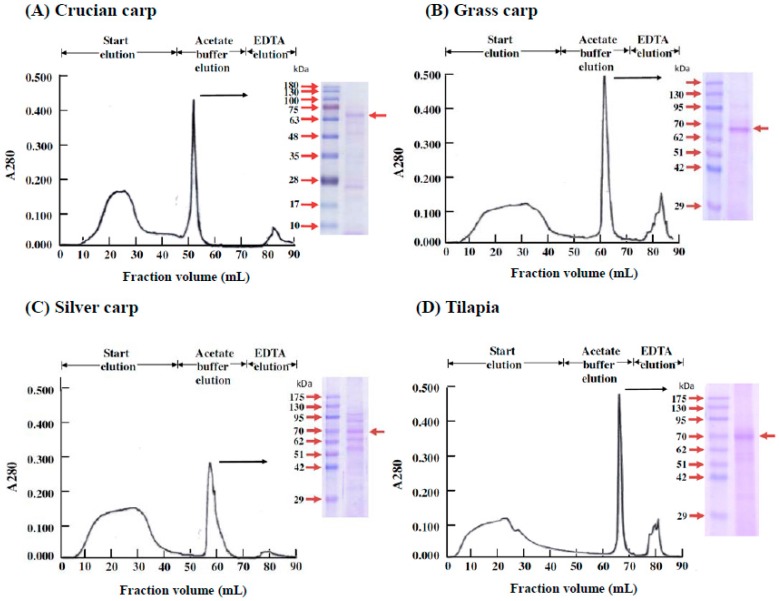
Isolation of the fish serum component stimulating the proliferation of new RBCs in head kidney cells of (**A**) crucian carp, (**B**) grass carp, (**C**) silver carp, and (**D**) tilapia. The head kidney cell suspension was supplemented with 10% serum from each fish species and ZnCl_2_ and then cultured for three days before being harvested. The harvested cells were separated into fractions 1 and 2 using a Percoll density gradient. The cells collected from fraction 1 were extracted using a detergent buffer, and the detergent extract was purified by Zn^2+^-IMAC. The acetate buffer eluates were pooled and analyzed by SDS-PAGE under reducing conditions, followed by staining with Coomassie blue to visualize the protein bands. The arrowhead denotes the position of the protein identified by nano-LC-MS/MS analysis as transferrin in crucian carp, grass carp, silver carp, and tilapia, respectively.

When common carp head kidney cells were cultured with supplemental ZnCl_2_ in the presence of carp serum, transferrin was identified as the active substance in fish serum that stimulates the proliferation of immature RBCs [1]. As shown in Figure 5, transferrin was confirmed as the active substance for all four fish species in the present study.

### 2.6. Effects of the Injection of ZnSO_4_ on Erythropoiesis in a Blood Loss Tilapia Model

**Figure 6:**
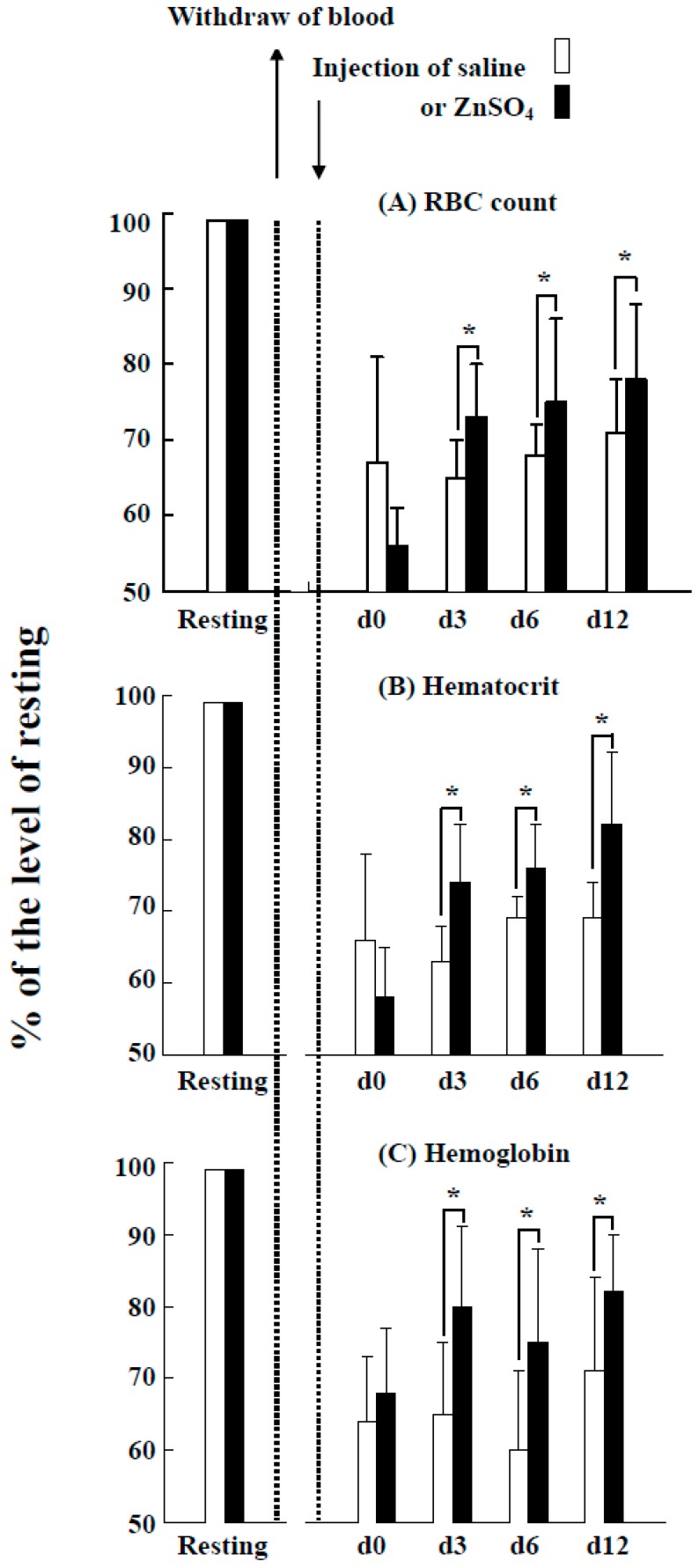
Effects of the injection of ZnSO_4_ on erythropoiesis in the blood loss tilapia model. At day zero, blood was withdrawn from the fish, and saline or ZnSO_4_ was injected. At day three, day six, and day 12, blood was sampled from the treated fish and analyzed. The data are expressed as the ratios of the RBC count (**A**), hematocrit (**B**) and hemoglobin (**C**) level at different days to the values at baseline. The results are the means ± SDs of three independent experiments. * Significant difference (*p* < 0.05) between the saline and ZnSO_4_ treatments.

**Figure 7:**
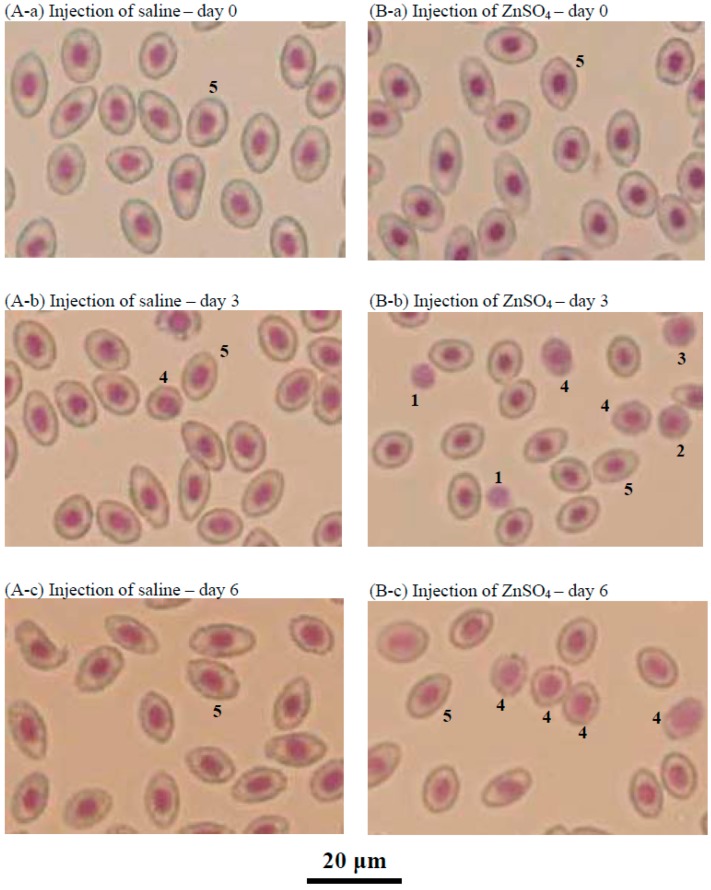
Giemsa staining of the blood cells sampled from tilapia subjected to blood loss and injected with saline (**A**) or ZnSO_4_ (**B**). At day zero, approximately 95% of the blood cells were stage 5 RBCs, erythrocytes (**A**-**a**,**B**-**a**). At day three and day six, the blood cell composition of the fish injected with saline (**A**-**b**,**A**-**c**) was similar to that on day zero; that is, approximately 95% stage 5 RBCs and 3% stage 4 RBCs (orthochromatic erythroblasts). However, for fish injected with ZnSO_4_, newly proliferated cells were observed on day three (**B**-**b**), such as stage 1 RBCs (lymphoid hemoblasts), stage 2 RBCs (early erythroblasts), and stage 3 RBCs (polychromatophilic erythroblasts). At day six, the blood cells from the fish injected with ZnSO_4_ included more stage 4 RBCs (orthochromatic erythroblasts) (**B**-**c**).

Two hours after the loss of approximately 20% of the blood in the tilapia, the RBC counts, hematocrit, and hemoglobin levels of the fish decreased to approximately 55%–65% of the baseline values (Figure 6). However, the group of tilapia injected with ZnSO_4_ exhibited significantly higher RBC counts, hematocrit values, and hemoglobin levels than the control group (injection of saline) after three days and until 12 days. Microscopic observation of the blood cells revealed few immature RBCs in the group injected with saline throughout the experimental period (Figure 7A-a,A-b,A-c). In contrast, immature RBCs of different stages were observed three and six days after injection of ZnSO_4_ (Figure 7B-b,B-c). These results indicate that excess in vivo zinc induced erythropoiesis in fish in three days. At day six, the blood cells from the fish injected with ZnSO_4_ included more stage 4 RBCs (orthochromatic erythroblasts) (Figure 7B-c).

## 3. Discussion

The common carp has extraordinarily high zinc levels in digestive tract tissues (~300 μg/g tissue) compared to other fish (~20 μg/g tissue), as first reported more than 40 years ago [13]. The physiological relevance of this high level of zinc was not clear until recently [1,9,11,14]. At low oxygen concentrations, zinc is released from the digestive tract tissue of the common carp and used as a signal to stimulate erythropoiesis in the head kidney of the fish [1]. Whether the use of zinc as a signal to induce erythropoiesis was unique to this fish species was unclear, but the present report indicates that this type of “zinc signaling” pathway also exists in other fish if a sufficient amount of zinc is sent to the head kidney cells.

Despite the variety of the four fish studied (crucian carp, grass carp, and silver carp belong to the *Cyprinidae* family, but the tilapia are in a completely different family, *Cichlidae*) and the different zinc levels (crucian carp have zinc levels as high as ~500 μg/g fresh tissue, but grass carp, silver carp, and tilapia all have zinc levels of ~20 μg/g fresh tissue), the stimulation of erythropoiesis by zinc was dose-dependent, with a significant activation concentration of 0.2–1.2 mM and maximum cell growth of 300%–600% (Figure 2). In vitro, zinc could be used as a signal to induce blood cell formation in fish. In the blood, the zinc is carried by transferrin (Figure 5).

An experimental blood loss model has been established in several fish species [15,16,17]. The response to blood loss appears to depend on the fish species: some species recovered in several days [15], whereas others require weeks [16,17]. In the present study, the bled tilapia exhibited no significant stimulation of erythropoiesis in 12 days (Figure 6, control group). However, the ZnSO_4_ injection group exhibited increased RBC, hematocrit and hemoglobin levels. These values decreased to 55%–65% of baseline in the control group but only 70%–80% of baseline in the ZnSO_4_ group at three days post-bleeding (Figure 6, ZnSO_4_ group). Microscopic observation of the blood of the ZnSO_4_-injected fish confirmed that many new immature RBCs proliferated (Figure 7). In vivo, the presence of additional zinc induced erythropoiesis in the bled tilapia.

In recent years, “zinc signaling” was reported to play a role in brain function, immunology, inflammation, growth control and bone diseases, and cancer [18]. To date, the use of zinc as a signal to stimulate erythropoiesis appears to only have been observed in the common carp [1,9,11]. Although it is not clear whether this mechanism also exists in other animals, the present report suggests a possible approach for the induction of erythropoiesis in animals through the supply of a certain additional level of zinc through either diet or injection. If zinc can act as a signal to trigger erythropoiesis in many fish species, this mechanism might also exist in a broader range of species. Mammalian red cells lack a nucleus and differ fundamentally from the red blood cells of fish, birds, and reptiles. Erythropoiesis occurs in the head kidney in teleost fish but in the bone marrow in mammals. We recently studied whether zinc could be used as a signal to induce red blood formation in rat bone marrow cells, and our results suggest that at certain levels, zinc could trigger erythropoiesis in mammals (unpublished paper). Anemia is a global human health concern, and the use of zinc as a signal to induce red blood cell formation in patients through zinc supplementation might be a possible treatment. Thus, the special feature of using zinc as a signal to stimulate red blood formation in the common carp might have implications for human health.

## 4. Materials and Methods

### 4.1. Fish

Cultured live fish (crucian carp, grass carp. silver carp, and tilapia) were obtained from a local fish farm and housed in a polyethylene tank in the laboratory for 2 months before the experiment [11]. The total length and total mass of the fish were: crucian carp, 22 ± 3 cm, 207 ± 69 g, (*n* = 44); grass carp, 41 ± 2 cm, 680 ± 101 g, (*n* = 6); silver carp, 39 ± 3 cm, 556 ± 73 g, (*n* = 6); and tilapia, 27 ± 1 cm, 362 ± 43 g, (*n* = 15). Animal use was reviewed and approved by the institutional animal care and use committee at National Taiwan Ocean University (105-J29803; License No. 102072).

### 4.2. Preparation of Head Kidney Cell Suspensions and Fish Serum

Head kidney cell suspensions and fish serum were prepared from each different fish species as described previously [1].

### 4.3. Suspension Culture of Head Kidney Cells with or without ZnCl_2_ Supplementation

The head kidney cell suspension (approximately 60 × 10^6^ cells/mL) from each different fish species was cultured with or without ZnCl_2_ as described previously [1]. The cultures were incubated at 27 °C in an atmosphere of 5% CO_2_. After 0, 1, 2, 3, and 4 days, cells were harvested to measure the total cell number and subjected to separation by Percoll density centrifugation. Four independent experiments were performed.

### 4.4. Separation of Harvested Head Kidney Cells into Two Fractions by Discontinuous Percoll Density Centrifugation

The harvested head kidney suspension was separated by discontinuous Percoll centrifugation (2.0 mL of 1.020 g/mL Percoll and 6.0 mL of 1.070 g/mL Percoll, both in 0.15 M NaCl solution) into 2 fractions as reported previously [9]. The cell numbers of fractions 1 and 2 were measured with an electronic cell counter.

### 4.5. Effects of ZnCl_2_ on the Growth of Fraction 1 Cells from the Head Kidneys of Different Fish Species

Aliquots of 0.1 mL of head kidney cell suspension (60 × 10^6^ cells/mL) from different fish species were added to equal volumes of DMEM/F12 medium supplemented with 20% fish serum from the respective fish species and different concentrations of ZnCl_2_. Zinc concentrations in the control groups were determined, and found to be about 0.01 mM. The cells were cultured as described above. At day 0 and 1, the cells were harvested and separated by Percoll density centrifugation. The cell numbers in fraction 1 were counted. Cell growth was expressed as the ratio of the fraction 1 cell number to that at the start of culture relative to the control. Six independent experiments were performed.

### 4.6. Microscopic Observation of Head Kidney Cells or Tilapia Blood by Giemsa Staining

The harvested head kidney cells or tilapia blood were directly fixed on slides, stained with the Giemsa method [19], and subjected to microscopic observation. RBCs were identified according to previous reports [3,12,20].

### 4.7. Immunofluorescence Staining of the Transferrin Receptor in Fish Head Kidney Cells

The fish head kidney cells were subjected to immunofluorescence staining of the transferrin receptor as previously reported [1] and were observed through fluorescence microscopy.

### 4.8. Isolation of the Active Substance in Fish Serum That Stimulates the Proliferation of Fraction 1 Cells in Fish Head Kidneys

Preparation of proliferated cells, detergent extraction of zinc-binding proteins from fraction 1 cells, immobilized metal affinity chromatography (IMAC) purification of zinc-binding proteins, electrophoresis, and nano-LC-MS/MS were performed as reported previously [1].

### 4.9. Experimental Blood Loss in Tilapia and Injection of ZnSO_4_ Solution

In each experiment, 54 acclimated tilapias were used. Before the experiment, 6 fish were removed, and 0.2 mL of blood was sampled from resting fish as the baseline. The remaining 48 fish were subjected to experimental blood loss by withdrawal (caudal venipuncture) of 3.0 mL blood from each fish, which represented approximately 20% blood loss because preliminary experiments indicated that the extractable blood of the tilapia was approximately 15 mL. After bleeding, the fish were allocated to two groups of 24 fish. Group A (control group) was injected with saline solution (0.25 mL), and Group B (experimental group) was injected with (1.47 mg/mL, or 5.1 mM) ZnSO_4_ solution (0.25 mL). The fish were returned to the culture tank, and no feed was given during the experimental period. Two hours after the injection of saline or ZnSO_4_ solution, 0.2 mL of blood was sampled from each fish in each group (6 fish) as blood from day 0. At days 3, 6, and 12, 0.2 mL of blood was sampled from 6 fish in each group. The collected blood was used for blood analysis and microscopic observation. Three different experiments were performed.

### 4.10. Blood Analysis

At each sampling time, the hematocrit, hemoglobin, and total RBC counts were determined using an auto hematology analyzer (Excell 500; Danam Electronics, Dallas, TX, USA) as reported previously [9]. Blood smears were microscopically observed.

### 4.11. Data Analysis

The data are expressed as the means ± standard deviations (SDs). The statistical significance of experimental results was calculated by one-way analysis of variance, followed by the least significant difference post hoc test, using SPSS 10.0 (SPSS Inc., Chicago, IL, USA).

## 5. Conclusions

Zinc is an essential element in organism, it has many function in life. In nature, the common carp has a unique way to use it as a signal to stimulate red blood cell formation by having a specific zinc-binding protein. Many fish do have no this specific zinc-binding protein, but the present report indicated that supplying a specific additional level of zinc exogenously could also induce erythropoiesis. It is very possible that this mechanism also exist in other animals including human beings. The potential of using this mechanism to treat anemia in human beings is high. However, how zinc stimulates erythropoiesis in fish (and may be in other animals) must be further investigated.

## Abbreviations

EPO	Erythropoietin
IMAC	Immobilized metal affinity chromatography
